# Understanding the Relationship Between the Big Five Personality Traits and the Cognitive Appraisals Leading to Emotions: An Integrative Narrative Review

**DOI:** 10.1177/17540739251372161

**Published:** 2025-09-26

**Authors:** Livia Sacchi, Elise Dan-Glauser

**Affiliations:** Institute of Psychology, 27213 University of Lausanne, Lausanne, Switzerland; Institute of Psychology, 27213 University of Lausanne, Lausanne, Switzerland

**Keywords:** emotion, appraisal, Big Five

## Abstract

In the Component Process Model (CPM), emotion is defined as a rapid psychological process whose emergence relies on appraisals (i.e., cognitive evaluations) of the environment. Personality encompasses many affective components and traits thought to shape specific appraisals, which in turn drive subsequent emotional outcomes. Despite the relevance of this relationship, it has mostly been studied within stress research. Therefore, this article aims at: (1) synthesizing prior research on the relationship between personality and appraisal, using the Big Five personality trait taxonomy as an integrative model and the alternative appraisal tradition of the CPM, and (2) providing an explanatory perspective on the nature of such relationships. The importance of understanding the mechanism linking individual differences and emotion responses is discussed.

## Introduction

### Emotions and Appraisal Processes

Emotions represent a fundamental aspect of the human condition. A wide range of emotions are experienced very frequently throughout the day (Zelenski & Larsen, 2000) and life (Carstensen et al., 2000). According to most emotion theories, an emotion is not a state but rather a rapid, multifaceted psychological process (Moors, 2010, 2013). This phenomenon emerges in response to changes in the environment, leading to the development of patterned responses (Scherer, 2005), modifying behaviors and fostering adaptation (Gross, 2014). Moreover, the dynamic unfolding of an emotional episode typically involves coupled changes in five components, namely: a cognitive component (i.e., appraisal); a feeling component (i.e., the subjective experience of the emotion); a motivational component (i.e., action tendencies); a somatic component (i.e., physiological activation); and, finally, a motor component (i.e., expressive behavior; Scherer & Moors, 2019).

As the cognitive component of an emotional episode, appraisal initiates the emotional unfolding through the assessment of a given internal or external situation, such as a memory or an encounter. In this way, appraisal evaluates the situational relevance in relation to the well-being and goals of the individual concerned (Ellsworth & Scherer, 2003), based on the subjective meaning attributed to it (Gross, 2014). Appraisal theories emphasize the fundamental role that appraisal plays, not only in the elicitation (i.e., causation) and differentiation of emotions (Scherer & Moors, 2019), but also in conditioning the intensity and the quality of the other emotional components (Moors et al., 2013). Finally, appraisal is thought to determine the type of emotion that is ultimately experienced (Wranik & Scherer, 2010).

The first wave of appraisal theories, initially rooted in stress research, can be traced back to the 1960s (Schorr, 2001). In the Transactional Model of Stress and Coping, Folkman and Lazarus (1984) distinguished between primary appraisals, which evaluate the significance of the stressor against its potential gains (challenges) or losses (threats), and secondary appraisals, which evaluate the individual's ability to cope with future consequences (Schorr, 2001). However, the concept of appraisal was later refined by Lazarus in the 1980s to extend and integrate the model with the new, emerging appraisal theories in the field of emotion, defined by Schorr (2001) as “modern.”

**Figure 1.**
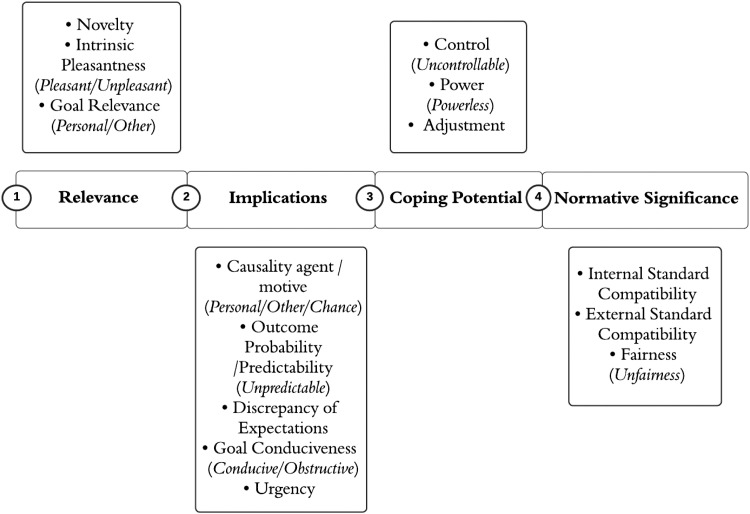
Multilevel appraisals, functional categories, and related checks and subordinate checks.

*Note.* The middle bold labels indicate the four functional categories of Relevance, Implications, Coping Potential and Normative Significance. Boxes below and above include their related Stimulus Evaluation Checks (SECs) and subordinate checks. Redrawn from Evidence of emotionantecedent appraisal checks in electroencephalography and facial electromyography by Coutinho et al. (2018). Licensed under CC BY 4.0 (https://creativecommons.org/licenses/by/4.0/). Changes were made by the authors.

Among modern appraisal theories, Scherer's (1984, 2001, 2009) Component Process Model (CPM) of emotion is considered as one of the most comprehensive. It provides a detailed, structural description of appraisal. In particular, the CPM posits the existence of a sequential and defined set of criteria responsible for situational appraisal, known as Stimulus Evaluation Checks (SECs), orchestrated by the four functional categories of Relevance, Implications, Coping Potential and Normative Significance (Scherer, 2009; Figure 1). Briefly, the functional category of Relevance regroups those SECs that evaluate the stimulus as either novel or familiar, pleasant/unpleasant, and relevant or irrelevant to the individual's current or long-term goal. The functional category of Implications regroups those SECs that evaluate the agents responsible for the occurrence of the event, whether this action is likely to produce (un)desirable consequences and if the situation is conducive or obstructive to personal goals. The functional category of Coping Potential regroups those SECs that evaluate an individual's perception of their ability to manage a given situation and their ability to cope with the expected consequences. Finally, the functional category of Normative Significance regroups those SECs that evaluate the meaning of the event in terms of compatibility with the individual's self-concept, as well as with social norms, and moral and legal values (Scherer, 2001). To streamline our terminology, we will henceforth use *appraisal* to denote SECs and their related subordinate checks (e.g., the Goal Relevance SEC can be differentiated into personal and other-oriented relevance of goals, which represent subordinate checks).

### Personality and Emotions: Extant Evidence on Their Interconnection

The extensive array of criteria inherent to modern appraisal theories is able to explain the multifaceted nature of emotional experience, from its elicitation to its differentiation (Scherer & Moors, 2019). Another layer of nuance is introduced when individual differences are considered. Indeed, people often report divergent emotions in response to the same situation—a well-documented phenomenon with extensive theoretical and empirical support. Appraisal content and frequency (Scherer, 2020; van Reekum & Scherer, 1997), as well as the strength of the appraisal–emotion relation (Kuppens & Tong, 2010; Silvia, 2008), vary substantially between individuals. Some authors suggest that these individual differences are driven by emotional dispositions, conceptualized as systematic tendencies or cognitive biases (Kuppens & Tong, 2010; Scherer, 2020). We believe these are part of what we define as personality, thereby signifying that individual differences in emotion processes can be attributed to personality variations.

The term “Personality” is derived from the Latin word “Persona,” which translates as “Mask” (Millon et al., 2004). It could be argued that there are as many personalities—and masks—as there are people in the world. However, within the scientific literature, three to six personality dimensions have consistently emerged over time, with the “Big Five” structural model of personality finding large consensus (John & Srivastava, 1999; Revelle & Scherer, 2009; Thalmayer et al., 2011). These five factors are: (a) Neuroticism, or Emotional (In)Stability, the tendency to experience negative affect; (b) Extraversion, the tendency to experience positive affect and socialize; (c) Openness/Intellect, the tendency toward curiosity and exploration; (d) Agreeableness, the tendency toward altruism and tenderness; and (e) Conscientiousness, the tendency toward discipline and diligence.

As these concise definitions illustrate, a strong interconnection exists between the Big Five traits and emotions. Indeed, a personality *trait* reflects the stable tendency to feel, behave, think, and desire in a consistent manner, over time and across different contexts (Revelle & Scherer, 2009), and thus characterizes how people “are” on average, including in the emotional sphere (Kuppens & Tong, 2010). Conversely, an emotion is generally referred to as a *state*, due to its episodic and transient nature, happening in the here and now (Scherer, 2009). It can thus be hypothesized that this state process is orchestrated by more stable constructs, such as personality (Revelle & Scherer, 2009). Indeed, the associations between Neuroticism and Negative Affect, and Extraversion and Positive Affect, respectively, are among the most well-established and replicated findings in the affective and personality literature (Reisenzein et al., 2020; Revelle & Scherer, 2009; Wilt & Revelle, 2017). More specifically, Neuroticism has been repeatedly linked to unhappiness and emotional distress, and identified as a risk factor for affective disorders, such as depression and anxiety (Tackett & Lahey, 2017), while Extraversion has been generally linked to assertiveness, sociability, energetic attitude, and happiness (Wilt & Revelle, 2017). Regarding Agreeableness and Openness, the former is mainly related to positive emotionality toward others, and to positive emotions such as compassion, love, and joy, while the latter is related to curiosity, awe, love, and amusement (Reisenzein et al., 2020). The affective component of Conscientiousness is less straightforward, given its preponderantly behavioral nature (Reisenzein et al., 2020; Revelle & Scherer, 2009). In examining this relationship, attention should not be given exclusively to the link between personality and emotion responses, but also to that between personality and appraisal.

### Personality and Emotions: A Role for Appraisal?

As demonstrated above, personality plays a special role in emotion-related outcomes, something that was already articulated three decades ago by Smith and Lazarus (1990) in their cognitive-motivational-emotive system model. It is of great interest to note that, at that time, the authors had already recognized personality as a plausible key factor in appraisal variability. Specifically, personality—operationalized in the model as a compound of needs, commitments, goals, knowledge, attitudes, and beliefs—interacts with situational construal (i.e., individual beliefs and knowledge about the situation, which are conditioned by objective situational features) by affecting appraisal processes. Subsequent refinements of this model led to further emphasis on the process-based and relational nature of appraisal. Appraisal was then hypothesized not only to rely on automatic cognitive mechanisms through which an emotional reaction could be speeded up, but also to entail constant transactions between the situational context and individuals’ needs and goals (Smith & Kirby, 2001; Smith & Kirby, 2009). Based on these accounts, there has been a recent resurgence of interest in the study of appraisal as one of the intermediary processes implicated in the causal chain linking personality to broader life outcomes, such as happiness, satisfaction or mental health (Keltner & Shiota, 2021; Poluektova et al., 2023; Scherer et al., 2022). Altogether, it seems that personality variations may serve as a plausible source of individual differences in both emotion responses and appraisals.

### Review Aim and Scope

In essence, personality reflects the predisposition to feel certain emotions more frequently, and it is deeply intertwined with the emotion realm (Revelle & Scherer, 2009). Similarly, both emotion and appraisal are influenced by individual differences—such as personality traits (Kuppens & Tong, 2010)—and emotions themselves depend on cognitive appraisal. Additionally, a substantial body of evidence has accumulated regarding the impact of personality on emotionality. However, this does not apply to research on the impact of personality on situational appraisal. Despite the extensive and renewed interest that appraisal has received as the pivotal element in emotion elicitation, differentiation, and possibly affecting distal, affect-related life outcomes (Keltner & Shiota, 2021; Reisenzein et al., 2020; Scherer et al., 2022), there is a lack of unified understanding of the interrelation between personality and appraisal.

We aimed to fill this intersectional research gap in two ways. First, we wanted to provide an up-to-date overview of the relationship between personality and appraisal, outside of stress research and within the CPM tradition. A number of publications addressed the issue, but these either were not intended as reviews (Kuppens & Tong, 2010), or solely endorsed Folkman and Lazarus's (1984) stress framework (Kilby et al., 2018). In order to achieve this goal, the Big Five (John & Srivastava, 1999; McCrae, 2020) was selected as the overarching framework for contextualizing the available personality–appraisal associations. This choice was inspired by the fundamental work of Bainbridge et al. (2022), who recently highlighted the widespread tendency in psychology to employ a multitude of analogous individual difference scales to predict a range of life outcomes. The authors demonstrated how several of the most frequently cited scales—measuring (affective) constructs such as trait self-esteem, anger, worry, impulsivity, aggression, perseverance, and compassion—appear to share a substantial amount of variance with domains and facets belonging to the Big Five taxonomy. In other words, when these scales are used to predict a given outcome over and above the Big Five, the incremental validity is minimal or comparable to the Big Five facets, indicating redundancy with the Big Five framework (Bainbridge et al., 2022). The extensive use of and reference to emotion trait scales such as trait anxiety, trait anger, trait sadness, or trait self-efficacy in emotion research (Poluektova et al., 2023; Scherer, 2009) substantiate the prevalence of the phenomenon identified by Bainbridge et al. (2022) in this field. It is imperative to emphasize the Big Five as an integrative model to propel advancements in both personality and emotion research. This approach fosters the synthesis of analogous findings, facilitates effective communication, and ultimately paves the way for a shared language among scholars (Bainbridge et al., 2022). The second aim of the present work is to provide an explanatory perspective that goes beyond merely reporting associations found in the reviewed literature, and clarifies how personality influences appraisal and subsequent emotion responses. To that end, we build on a recent summary by Reisenzein et al. (2020), who highlighted three plausible mechanisms accounting for interindividual differences in appraisal.

## Methods

### Review Approach

The selection of studies for this review was conducted through a search in Web of Science and PsycInfo between February 2021 and June 2025. The complete list of search terms, available in the Supplemental Material, includes keywords related to the concepts of interest, such as (cognitive) appraisal, Big Five, personality, and emotion. Following a thorough review of the titles and abstracts, and the removal of duplicates, 41 studies were selected and screened against a series of basic inclusion and exclusion criteria.

Because our focus was on work outside stress research, we excluded articles that based their conceptual framework on Folkman and Lazarus's (1984) stress model, thus referring to primary or secondary appraisals. Conversely, we retained articles that endorsed modern appraisal theories (Schorr, 2001) as their methodological framework, or in their formulation/assessment of appraisal. With respect to personality, we included studies assessing the Big Five traits, whether they measured a single dimension or several, up to all five, regardless of the assessment instruments used—e.g., Big Five Inventory (Rammstedt & John, 2007); the International Personality Item Pool (Goldberg et al., 2006); the Revised NEO Personality Inventory and the NEO-Five Factor Inventory (Costa & McCrae, 1992); the Ten-Item Personality Inventory (Gosling et al., 2003); or the Big Five Aspect Scale (DeYoung et al., 2007). In our search, we also encountered studies that only partially met our inclusion criteria. These studies either endorsed the Big Five personality framework, but revisited appraisal definitions and constructs, or employed appraisal as in modern appraisal theories, but relied on pooled measures of personality traits or proxies for the Big Five traits. We still deemed these studies relevant to be cited as they proved how findings from conceptually similar scales can be integrated under the Big Five framework (Bainbridge et al., 2022). These additional studies were however reviewed in dedicated subsections named “Other Studies.”

Following the selection process, a total of 15 studies were retained for our review. Results are organized by examining each of the Big Five traits in relation to the four functional categories of Relevance, Implications, Coping Potential, and Normative Significance, and to their corresponding SECs and subordinate checks. Based on the existing literature on personality and stress/affect (Kilby et al., 2018; Reisenzein et al., 2020; Revelle & Scherer, 2009), some hypotheses could be made regarding the nature and direction of associations. Individuals higher in Neuroticism tend to interpret events as more negative—regardless of the situation intrinsic negativity—and report lower self-esteem (Mu et al., 2019; Suls & Martin, 2005). Therefore, Neuroticism is expected to be positively associated with the appraisals of Unpleasantness, Unfairness, and Uncontrollability. Because of its domineering, assertive, and reward-seeking nature (Wilt & Revelle, 2017) and its strong link with Positive Affect, Extraversion is expected to show positive associations with the appraisals of Pleasantness, Agency, Goal Conduciveness, and Control/Power. Openness/Intellect, characterized by esthetic interests, curiosity, novelty seeking, and intelligence (John & Srivastava, 1999; Sutin, 2017), is expected to be positively associated with the appraisals of Novelty, Pleasantness (driven by an intrinsic motive of discovery), and Goal Conduciveness (driven by overcoming obstacles with creativity). Agreeableness, characterized by tenderness and altruism (Graziano & Tobin, 2017), is expected to be positively related to the appraisals of Pleasantness, Relevance, Agency, and negatively to Other-Blame. Finally, given the sparse evidence concerning the affective nature of Conscientiousness (Reisenzein et al., 2020), and its diligent, goal-oriented and disciplined nature, it was hypothesized that this trait would be positively related to the appraisals of Control/Power and of Internal and External Standard Compatibility.

## Results

Table 1 presents the main characteristics of interest of the 15 retained studies. Given the pivotal function of appraisal in emotion generation, where available, we highlighted the reported effects of appraisal on resulting emotions and/or emotion components. As expected, the findings were more abundant for the traits of Neuroticism and Extraversion, given their well-documented robust association with the affective domain. However, interesting results emerged also for the other traits, albeit less diversified. Of note, the functional categories of Coping Potential and Normative Significance were the least endorsed—or researched upon—in relation to the traits of Openness, Agreeableness and Conscientiousness. Table 2 provides a visual overview of the results, along with the respective effect sizes when available. Due to the heterogeneity of the coefficients reported, the results are not directly comparable. We therefore proceed by listing the results, and by commenting on the most salient appraisals per trait in the Discussion section.

**Table 1.** Selected Studies on Appraisal and the Big Five.

	Authors		Sample		Delivery Format		Appraisal framework		Personality measurement		Emotion induction method		Effects on emotional outcomes?	
	Inglis et al. (2018)		*N* = 192 community adults 69% women *M*_age_ = 26.33 (*SD* = 11.86)		Online survey		Modern appraisal theories (Ellsworth & Smith, 1988; Frijda et al., 1989; Roseman et al., 1996; Lazarus, 2001; Scherer, 2001)		Affiliative/agentic extraversion (enthusiasm/assertiveness in the BFAS; DeYoung et al., 2007)		Affiliative family imagery		↑ Affiliative extraversion → ↑ Intrinsic pleasantness → ↑ Warmth-affection ↑ Affiliative extraversion → ↑ Internal standard compatibility → ↑ Warmth-affection ↑ Affiliative extraversion → ↑ Relevance → ↑ Positive activation	
	Mohammadi and Vuilleumier (2020)		*N* = 638 online community workforce 56% male *M*_age_ = 34 (*SD* = 11)		Online survey on crowdsourcing platform		CPM (Scherer, 2009) : emotion components measured with the GRID instrument (Fontaine et al., 2013)		BFI-10 (Rammstedt & John, 2007)		Emotionally engaging video-clips			
	Sacchi and Dan-Glauser (2025)		*N* *=* 500 university students 83% women *M*_age_ = 22.41 (*SD* = 3.23)		Online survey		CPM (Scherer, 2009) : Appraisal component measured with the CoreGRID (Scherer & Fontaine, 2013) ; Experience and autonomic arousal components measured with the MiniGRID (Scherer et al., 2013)		French version of the NEO-FFI (Costa & McCrae, 1992; Rolland et al., 1998)		Slightly ambiguous social scenarios: one negative, adapted in French from Zimmer-Gembeck and Nesdale (2013); one positive, adapted in French from Farrell et al. (2015) and Rohrbacher and Reinecke (2014)		Negative scenario: ↑ Neuroticism → ↑ Negative Consequences → ↑ Experiential activation ↑ Neuroticism → ↑ Powerlessness → ↑ Experiential and physiological activation ↑ Neuroticism → ↓ Consequences adjustment → ↑ Experiential and physiological activation Positive scenario: ↑ Neuroticism → ↓ Confirmed expectations → ↓ Experiential and physiological activation ↑ Neuroticism → ↑ Powerlessness → ↑ Experiential and physiological activation ↑ Extraversion → ↑ Pleasantness → ↑ Experiential activation ↑ Agreeableness → ↓ External standards Incompatibility → ↑ Experiential activation ↑ Conscientiousness → ↓ Powerlessness → ↓ Experiential and physiological activation	
	Scherer (2020)		*N* *=* 190 participants from a larger panel study (study 2) 49.2% women *M*_age_ = 45.5 (*SD* = 12.2)		Online study via qualtrics		Appraisal bias model (Mehu & Scherer, 2015) based on the CPM (Scherer, 2009)		Appraisals from the emotion disposition index (EMODI); TIPI (Gosling, Rentfrow & Swann, 2003); Drive subscale of the BAS (Carver & White, 1994); SoC (Ryser, Hosoya & Scherer, 2016)		Negative real-life scenarios		↑ Control/power → ↑ Coping potential → ↓ Worry / ↓ Sadness / ↓ Anger / ↑ Good humor ↑ Conscientious/agreeable → ↓ Valence → ↑ Worry / ↑ Sadness / ↓ Good humor ↑ Conscientious/agreeable → ↑ Relevance → ↓ Good humor	
	Scherer et al. (2022)		Study 1— Wave 14 of the Swiss household panel (SHP; Tillmann et al., 2022), *N* *=* 4,859, 55% women, age range = 18-65		Computer-Assisted Telephone Interview		Appraisal bias model (Mehu & Scherer, 2015) based on the CPM (Scherer, 2009)		Appraisal biases score of low coping potential obtained via CFA of two scales on coping (Pearlin & Schooler, 1978) and control (Lachman & Weaver, 1998). Extraversion and neuroticism from the BFI-10 (Rammstedt & John, 2007)		Self-report questionnaires		↑ Neuroticism → ↑ Low coping potential → ↑ Emotion disposition for sadness and Worry → ↑ Risk for mood disorders (depression/anxiety) ↑ Extraversion → ↓ Low coping potential → ↓ Emotion disposition for sadness and worry	
	Tong (2010)		*N* *=* 384 university undergraduates 63% women *M*_age_ = 22.08 (*SD* = 1.36)		EMA		Modern appraisal theories (Scherer, 1997; Smith & Ellsworth, 1985; Tong et al., 2007)		Neuroticism scale from the 10-item IPIP scale (Goldberg, 1992)		Naturally occurring daily life events		↑ Neuroticism: • ↓ Intrinsic pleasantness / ↓ Fairness × anger ↑ • ↑ Obstacles × anger ↑ • ↓ Intrinsic pleasantness / ↓ Control × sadness ↑ • ↑ Obstacles/ ↑ Fairness × sadness↑ • ↓ Predictability / ↓ Control × fear ↑ • ↓ Pleasantness/ ↑ obstacles / ↑ Fairness × fear ↑ • ↓ Intrinsic pleasantness / ↑ Obstacles × guilt ↑ • ↓ Fairness × guilt ↑	
	Tong et al. (2006)		*N* *=* 118 police officers 100% male *M*_age_ = 27.3 (*SD* = n/a)		EMA		Modern appraisal theories (Tong et al., 2005)		NEO-PI-R (Costa & McCrae, 1992)		Naturally occurring daily life events			
	Wilt (2014) (unpublished doctoral dissertation)		*N* = 91 participants 79% women *M*_age_ = 26.46 (*SD* = 8.46)		Online/text-messaging experience sampling protocol for 1 week		Appraisal theories of situational perceptions (Nezlek et al., 2008; Scherer et al., 2006; Tong et al., 2009)		44-item BFI (John et al., 1991)		Text-messaging cards on affect, behavior, cognition and desire (ABCD) content of daily-life experiences, four times a day			
	Other studies													
	Cummings et al. (2019)		*N* = 120 first year undergraduate psychology students 74% women *M*_age_ = 22.75 (SD = 8.19)		Online survey on campus		Appraisals of pleasantness and significance from Warriner et al. (2013)		Conscientiousness: 60 items of the IPIP NEO-PI-R; Neuroticism and extraversion: 10 items each of the IPIP NEO-FFI (Goldberg, 1992)		Administration of 80 academic-related words of varying motivational valence			
	Silvia (2008)		*N* = 83 university students (Exp 1); 72.3% women N = 122 university students (Exp 2); 76.2% women *M*_age_ = n/a (SD = n/a)		Paper-and-pencil in group sessions		Appraisals of coping potential and interest taken from Silvia (2005), Berlyne and Peckham (1966), or Scherer (2001)		Trait curiosity factor out of three curiosity measures, including the 20-item openness to experience from the IPIP (Goldberg et al., 2006)		Exp1: Complex poems Exp2: Complex and simple pictures of artworks		Exp1 ↑ Trait curiosity → ↑ Coping potential → ↑ Interest Exp2 (Simple pictures) ↑ Trait curiosity → ↑ Coping potential (Complex pictures) ↑ Trait curiosity → ↑ Coping potential → ↑ Interest	
	Fayn, Tiliopoulos, et al. (2015)		*N* = 99 first-year university students 77% women *M*_age_ = 19.41 (SD = 3.48)		Online sessions on campus		Appraisals of novelty, interest and coping potential from Silvia (2005)		100-item BFAS (DeYoung et al., 2007): 10 items assessing openness; 10 items assessing Intellect		7 quotations from the Oxford dictionary		↑ Intellect → ↑ Coping potential → ↑ Interest	
	Fayn, MacCann, et al. (2015)		*N* = 225 university students (Study 2) 69% women *M*_age_ = 20.56 (SD = 4.91)		Online sessions on campus: groups of 1–8 participants		Appraisals of novelty and coping potential from Silvia (2005)		100-item BFAS (DeYoung et al., 2007): 10 items assessing openness; 10 items assessing intellect		Administration of 18 visual art stimuli		↑ Openness × novelty: ↑ Interest ↑ Intellect × novelty: ↑ Interest ↑ Openness × novelty: ↑ Pleasure	
	Fayn et al. (2017)		*N* = 191 university students 77.5% women *M*_age_ = 19.27 (SD = 3.70)		Online sessions on campus: groups of 1–10 participants		Appraisals of novelty and coping potential taken from Silvia (2005)		100-item BFAS (DeYoung et al., 2007): 10 items assessing openness; 10 items assessing intellect		Administration of 30 stimuli (10 visual art; 10 science stories, 10 philosophical quotations)		For all types of stimuli: ↑ Openness → ↑ Coping potential → Interest For science and philosophy stimuli: ↑ Openness × novelty iInterest	
	Kuppens and Van Mechelen (2007)		*N* = 124 psychology students 50% women *M*_age_ = 18.9 (SD = n/a)		Paper & Pencil		Appraisals of other-blame, frustration and threat to self-esteem drawn from different appraisal theories; focus on transactional assumptions of emotions by Lazarus (2001)		Dutch versions of the NEO-FFI neuroticism and Spielberger trait anger scale (Defares, van der Ploeg & Spielberger, 1980).		Directed imagery task through situation vignettes		↑ Neuroticism: • ↑ Other-blame × someone else responsible ↑ Trait-anger: • ↑ Other-blame × omeone else responsible • ↑ Other-blame × self/circumstances responsible	
	Zajenkowski and Matthews (2019)		*N* = 224 (69.6% women) community volunteers *M*_age_ = 23 (SD = 5.96)		Online survey		Subjectively sssessed intelligence (SAI; Zajenkowski & Gignac, 2018) and satisfaction with intelligence (SI; Diener et al., 1985) framed as cognitive appraisal Lazarus (2001)		Polish version of the BFAS measuring with 10 items each openness and intellect assessed (DeYoung et al., 2007; Strus, Cieciuch & Rowiński, 2014)		Self-report questionnaires		↑ Intellect ↑ Understanding potential (SI) → ↑ Positive affect / Hedonic tone / Life satisfaction	

*Note.* ↑ = positive association; ↓ = negative association; − = no significant association; → = associated with. BAS = Behavioral Activation Scale; BFAS = Big Five Aspect Scale; BFI-10 = Big-Five Inventory-10; CFA = confirmatory factor analysis; CPM = component process model; EMA = ecological momentary assessment; IPIP *=* International Personality Item Pool; NEO-FFI = NEO-Five Factor Inventory; NEO-PI-R = The Revised NEO Personality Inventory; SoC = Sense of Control Scale; TIPI = Ten-Item Personality Inventory.

**Table 2.** Summary of Results.

		Neuroticism (10 studies)		Extraversion (8 studies)		Openness (11 studies)		Agreeableness (5 studies)		Conscientiousness (6 studies)	
	**RELEVANCE**										
											
	Relevance	↑ (.32, Cummings et al., 2019)^a^		↑ (.26, Inglis et al., 2018)^c^; ↑ (−.26, Cummings et al., 2019)^a^				↑ (.27, Scherer, 2020)^1,c^; Adjusted R^2^ (Including EC knowledge = .41)		↑ (.27, Scherer, 2020)^1,c^; Adjusted R^2^ (including EC knowledge = .41); ↑ (.42 /−.34 / .30 / .32, Cummings et al., 2019)^a^; Adjusted R^2^ = 0.30 (appraisals of academic stimuli predicting conscientiousness)	
	*Other-(Goal) Relevance*	− −		− −		− −		− −		− −	
	Novelty	−		−		↑↑ (Openness/Intellect as moderator of novelty-Interest: .12 / .12; openness as moderator of novelty-pleasure: .16; Fayn, MacCann et al. 2015)^c^; ↓ (Art stimuli: −.19, Fayn et al. 2017)^c^; − − −		↓ (≃ -.10, Mohammadi & Vuilleumier, 2020)^a^		−	
	*Interest*										
	(Intrinsic) pleasantness / valence	↓ (−.59, Tong et al., 2006)^b^; ↓ (−.07, Tong, 2010)^a^; ↓ (−.18 /−.35 /−.26, Cummings et al., 2019)^a^ − −		↑ (.22, Inglis et al., 2018)^c^ ; ↑ (.30 / .27, Cummings et al., 2019)^a^; ↑ (.19, pleasantness, positive scenario, Sacchi and Dan-Glauser, 2025)^c^ ; − − −		− − −		↓ (−.24, Scherer, 2020)^1,c^; Adjusted R^2^ (including EC knowledge = .38); ↑ (.28, Wilt, 2014)^a^; − −		↓ (-.24, Scherer, 2020)^1,c^; Adjusted R^2^ (including EC knowledge = .38); ↑ (.61, Tong et al., 2006)^b^; ↑ (.36 / .49 / .43, Cummings et al., 2019)^a^; Delta R^2^ = 0.09 (pleasantness appraisals of academic stimuli predicting conscientiousness, entered after relevance appraisals) − −	
	*Subjective unpleasantness*	↑ (≃ .10, Mohammadi & Vuilleumier, 2020)^a^		−		−		−		−	
	*Other-unpleasantness*	−		↓ (≃ -.10, Mohammadi & Vuilleumier, 2020)^a^		↑ (≃ .20, Mohammadi & Vuilleumier, 2020)^a^		−		↑ (≃ < .10, Mohammadi & Vuilleumier, 2020)^a^	
	**IMPLICATIONS**										
	*Cause/agency-self*	↓ (−.04, Tong, 2010)^a^ ; − −		− − −		↑ (.23, Wilt, 2014)^a^− −		↑ (.21, Wilt, 2014)^a^ − −		↑ (.23, Wilt, 2014)^a^; − −	
	*Cause/agency-other*	↑ (.09, Tong, 2010)^a^ − −		↓ (-.17, Scherer, 2020)^c^; Adjusted R^2^ (including EC knowledge = .09)−		↑ (.95, Tong et al., 2006)^b^−		−		↓ (−.97, Tong et al., 2006)^b^−	
	*Cause/agency-circumstances/chance*	− −		− −		↓ (≃−.20, Mohammadi & Vuilleumier, 2020)^a^−		− −		↓ (≃−.20, Mohammadi & Vuilleumier, 2020)^a^−	
	*Other-blame x agency-other*	↑ (.26 / Trait anger .43, Kuppens & Van Mechelen, 2007)^a^									
	Outcome probability	−		−		−		−		−	
	*Certainty*	↓ (−1.20, Tong et al., 2006)^b^−		− −		↑ (.28, Wilt, 2014)^a^−		− −		↑ (.88, Tong et al., 2006)^b^−	
	*Predictability*	↓ (−.10, Tong, 2010)^a^ −		−		−		−		−	
	*Unpredictability*	− −		− −		− −		↓ (≃−.10, Mohammadi & Vuilleumier, 2020)^a^ −		↑ (≃ .10, Mohammadi & Vuilleumier, 2020)^a^ −	
	Discrepancy of expectations/ confirmed expectations	↓ (−.11, confirmed expectations, positive scenario, Sacchi & Dan-Glauser, 2025)^c^		−		−		↓ (−.17, Scherer, 2020)^1,c^; Adjusted R^2^ (including EC knowledge = .02) −		↓ (-.17, Scherer, 2020)^1,c^; Adjusted R^2^ (including EC knowledge = .02) −	
	Goal conduciveness / negative consequences	↓ (−.58, Tong et al., 2006)^b^;↑ (.17, negative consequences, negative scenario, Sacchi & Dan-Glauser, 2025)^c^ −		↑ (.24, Inglis et al., 2018)^c^;↑ (.28, Wilt, 2014)^a^;− −		↑ (.20, Wilt, 2014)^a^ − −		↑ (.22, Wilt, 2014)^a^ − −		↑ (.31, Wilt, 2014)^a^− −	
	*Obstacles*	↑ (.15, Tong, 2010)^a^									
	Urgency										
											
	**COPING POTENTIAL**										
											
	Coping potential (power, coping, control)	↑ (.15, low coping potential, Scherer et al., 2022)^c^; Adjusted R^2^ (including other background variables = .34)		↓ (−.13, low coping potential, Scherer et al., 2022)^c^; Adjusted R^2^ (including other background variables = .34)							
	*Understanding ability/potential*					Openness ↑↑↑ (Art stimuli: .34 / indirect effect: .13 ;science stimuli: .13 / indirect effect: .09; philosophy stimuli: .35 / indirect effect: .15, Fayn et al. 2017)^c^;Trait curiosity ↑↑↑ (Complex poetry: .33 / indirect effect: .18; complex images: .34 / indirect effect: .12 ; simple images: .27, Silvia, 2008)^c^; Intellect ↑ (Quotes: .36 / indirect effect: .08, Fayn, Tiliopoulos, et al., 2015)^c^; ↑ (.62 /.47, Zajenkowski & Matthews, 2019)^a^− − − −					
	Control	−		− −		− −		↑ (.21, Wilt, 2014)^a^−		↑ (.22, Wilt, 2014)^a^	
	*Control-self*	↓ (−1.28, Tong et al., 2006)^b^−		−		−		−		↑ (1.31, Tong et al., 2006)^b^	
	*Control-circumstances*	↑ (.09, Tong, 2010)^a^									
	Power/ powerlessness	↑ (.21, powerlessness, negative scenario, Sacchi & Dan-Glauser, 2025)^c^ ↑ (.11, powerlessness, positive scenario, Sacchi & Dan-Glauser, 2025)^c^		−		−		−		↓ (−.17, powerlessness, positive scenario, Sacchi & Dan-Glauser, 2025)^c^	
	Adjustment	↓ (−.24, negative scenario, Sacchi & Dan-Glauser, 2025)^c^		−		−		−		−	
	Dominance	−		−		−		−		−	
	*Effort/exert*	↑ (.10, Tong, 2010)^a^−		−		−		−		−	
											
	**NORMATIVE SIGNIFICANCE**										
											
	Internal standard compatibility	−		↑ (.28, Inglis et al., 2018)^c^		−		−		−	
	*Incongruent with standards*	−		−		−		−		−	
	External standard compatibility *Violating moral/legal standards*	↑ (1.31, Tong et al., 2006)^b^− −		↓ (−1.01, Tong et al., 2006)^b^ − −		− − −		↓ (-.10, external standard incompatibility, positive scenario, Sacchi & Dan-Glauser, 2025)^c^ − −		↓ (−1.46, Tong et al., 2006)^b^− −	
	Fairness	↓ (−.02, Tong, 2010)^a^−		−		−		−		−	
	*Unfairness*	↑ (1.15, Tong et al., 2006)^b^		↓ (−.93, Tong et al., 2006)^b^		−		−		↓ (−1.11, Tong et al., 2006)^b^	
											

*Note*. ↑ = positive association; ↓ = negative association; − = no significant association. EC = emotional competence.

^1^ Conscientious/agreeable pooled variable.

^a^ Correlation coefficient.

^b^ Unstandardized regression coefficient.

^c^ Standardized regression coefficient.

## Neuroticism and Cognitive Appraisals

### Neuroticism and Relevance

In an ecological momentary assessment study conducted on a sample of 118 male Singaporean police officers, it was found that those with higher levels of Neuroticism were less likely to appraise events as pleasant (Tong et al., 2006). This finding was confirmed by a subsequent study focusing exclusively on Neuroticism in a student sample (Tong, 2010). Moreover, Neuroticism was found to significantly interact with cognitive appraisals in the experience of negative emotions: while experiencing the same decrease in Pleasantness as compared to subjects scoring lower on the trait, those higher in Neuroticism reported stronger increases in anger and sadness, and greater changes in guilt (Tong, 2010). In contrast, again in the context of ecological momentary assessments via online surveys and text-messaging, Wilt (2014) found no significant association between the Big Five Inventory Emotional Stability and the appraisal of Pleasantness at the aggregate state mean levels.

In their data-driven study on emotion components, Mohammadi and Vuilleumier (2020) additionally explored Big Five–appraisal partial correlations with large range heatmaps. Appraisals were formulated as in the GRID questionnaire, which operationalizes the CPM components (Fontaine et al., 2013). When viewing video clips of varying valence, Neuroticism correlated positively with the appraisal of Subjective Unpleasantness, but showed no significant relationship with Other-Unpleasantness, Other-Relevance, and Novelty (i.e., “Occurred Suddenly”; Mohammadi & Vuilleumier, 2020). In the context of two slightly ambiguous daily-life scenarios tailored to a student population, Sacchi and Dan-Glauser (2025) applied exploratory mediation analysis (Serang et al., 2017) to obtain a set of influential appraisals mediating the relationship between each Big Five trait and emotional reactivity. Personality was measured with the NEO-Five Factor Inventory, while CPM emotion components—similarly to Mohammadi and Vuilleumier (2020)—with a GRID-derived instrument (Scherer et al., 2013). In the positive scenario, which depicted the preparation of a birthday party, Neuroticism was unrelated to Pleasantness and Other-Relevance (Sacchi & Dan-Glauser, 2025).

#### Other Studies

Despite endorsing appraisal theories as the guiding framework, Cummings et al. (2019) revisited the appraisal of Pleasantness and of Relevance/Significance and assessed them on a scale ranging from 1 (unhappy, annoyed, melancholic; irrelevant/not significant etc.) to 9 (happy, pleased, satisfied; relevant/significant). Furthermore, the study used highly specific stimuli—namely emotionally charged academic-related words—which differ from those generally used in emotional induction procedures (i.e., vignettes, scenarios, and past experiences recall). Overall, Neuroticism correlated positively with the appraisal of Relevance/Significance, but not with that of Pleasantness, of academic-avoidance words (such as “failure”, “drop-out”, and “stupidity”). In contrast, the trait correlated negatively with the appraisal of Pleasantness, but not with that of Relevance/Significance, of academic-approach words (such as “capability”, “expertise”, and “intelligence”), performance-evaluation words (such as “test”, “mark”, and “grade”), and academic neutral words (such as “campus”, “semester”, and “university”).

### Neuroticism and Implication

In Tong (2010), Neuroticism significantly predicted the appraisals of Responsibility-Self and Responsibility-Others in negative and positive directions, respectively. However, in Tong et al. (2006), Neuroticism did not significantly predict the appraisals of Agency-Self, Agency-Others, and Agency-Circumstances. Similarly, Wilt (2014) and Mohammadi and Vuilleumier (2020) reported no significant associations with the appraisals of Agency and Chance-cause, respectively.

In Tong et al.’s (2006) study, participants with higher Neuroticism scores were significantly less likely to appraise situations as conducive to important goals (“Goal Conduciveness”) and certain (“Certainty”). Conversely, Wilt (2014) found no significant association between the Big Five Inventory Emotional Stability and these two appraisals (with the former formulated as “Goal-Achievement Expectancy”). In Tong's (2010) study, the trait was negatively correlated with the appraisal of Predictability, and positively with that of Obstacles. Specifically, when events were appraised as less predictable, and goals were hindered by obstacles, students scoring higher on Neuroticism experienced greater increases in anger, sadness, fear, and guilt. In contrast, two studies found no significant associations between the trait and the appraisals of Outcome Probability (“Predictable Consequences”) and Unpredictability (Mohammadi & Vuilleumier, 2020; Sacchi & Dan-Glauser, 2025).

In Sacchi and Dan-Glauser (2025), the appraisal of Negative Consequences (i.e., Goal Conduciveness) positively mediated the relationship between Neuroticism and the intensity and duration of the emotional episode. This relationship emerged in the negative, mildly ambiguous social scenario, in which rejection might have been inferred. In the positive scenario, Neuroticism predicted weaker experiential and physiological reactivity through lower appraisal of Confirmed Expectations (Sacchi & Dan-Glauser, 2025).

#### Other Studies

Kuppens and Van Mechelen (2007) explored the heterogeneity of anger experience by employing Neuroticism and its proxy Trait-Anger. The traits correlated positively with the appraisal of Other-blame. Interestingly, this relation was significant for Neuroticism only in situations where the responsibility was of someone else, whereas for Trait-Anger it did so regardless of the origin of the responsibility.

### Neuroticism and Coping Potential

Participants higher in Neuroticism were less likely to appraise events as being under their control (Tong et al., 2006). In Tong's (2010) study, this association was not significant but, compared to participants scoring lower on Neuroticism, those scoring higher showed greater increases in sadness and fear when they perceived situations to be less under their control (“Control-Self”). In contrast, they reported no change in these emotions when situations were perceived as dependent on circumstances (“Control-Circumstances”). While Wilt (2014) found no significant association between the Big Five Inventory Emotional Stability and appraisal of Control, in Scherer et al. (2022), Neuroticism was a significant predictor not only of low Coping Potential appraisal bias, but also of the emotional dispositions of Sadness/Worry and of mood disorders risk. The authors therefore hypothesize a mediating mechanism for the low Coping Potential appraisal bias.

In the negative scenario of Sacchi and Dan-Glauser's (2025) study, the appraisals of Powerlessness and Consequences Adjustment positively mediated the relationship between Neuroticism and the intensity and duration of the emotional episode, as well as physiological reactivity, again assessed with a GRID-derived instrument (Scherer et al., 2013). Interestingly, the appraisal of Powerlessness also emerged as an influential mediator in the Neuroticism–experience and Neuroticism–autonomic arousal associations in the positive scenario.

Finally, results for the appraisal of Effort were mixed: Tong et al. (2006) found a nonsignificant relationship with Neuroticism, whereas Tong (2010) reported a positive one.

### Neuroticism and Normative Significance

Whereas in Tong et al. (2006) officers with higher Neuroticism scores were significantly more likely to appraise situations as unfair (“Unfairness”) and violating moral standards (“Moral Violation”), in Mohammadi and Vuilleumier (2020) and Sacchi and Dan-Glauser (2025) these results were not confirmed for the appraisals of Internal and External Standards Incongruence. Tong (2010) found a significant negative association with the appraisal of Fairness. Moreover, moderation analyses revealed that, upon decreases in perceived Fairness, participants higher in Neuroticism reported stronger increases in anger, fear, sadness, and guilt (Tong, 2010). Again, Wilt (2014) found no significant association between the Big Five Inventory Emotional Stability and the appraisal of Fairness.

### Extraversion and Cognitive Appraisals

#### Extraversion and Relevance

Inglis et al. (2018) focused on Affiliative Extraversion—also termed Enthusiasm—which corresponds to the “Warmth” facet of the Revised NEO Personality Inventory. This trait significantly predicted the appraisals of Intrinsic Pleasantness and Importance (i.e., Relevance). More interestingly, the authors found that the former appraisal significantly mediated the relationship between Affiliative Extraversion and the emotional state of warmth-affection, while the latter significantly mediated the relationship between the trait and positive emotional activation. Similarly, only in the slightly ambiguous positive scenario of Sacchi and Dan-Glauser's (2025) study, the appraisal of Pleasantness positively mediated the relationship between Extraversion and the intensity and duration of the emotional episode.

However, four studies reported no significant association between Extraversion and the appraisals of Pleasantness, Subjective Unpleasantness, Other-Relevance, and/or Novelty (Mohammadi & Vuilleumier, 2020; Sacchi & Dan-Glauser, 2025; Tong et al., 2006; Wilt, 2014). Interestingly, in Mohammadi and Vuilleumier (2020), the trait was marginally and negatively correlated with the appraisal of Other-Unpleasantness.

#### Other Studies

Extraversion was positively and significantly correlated with the appraisal of Pleasantness, but not that of Relevance, of academic-approach and performance-evaluative stimuli. In contrast, this trait was found to be significantly and negatively correlated with the appraisal of Relevance, but not that of Pleasantness, of academic-avoidance stimuli (Cummings et al., 2019).

#### Extraversion and Implications

While in Wilt (2014) and in Inglis et al. (2018) Extraversion was positively correlated with the appraisal of Goal Conduciveness, this was not the case in Tong et al. (2006). Mohammadi and Vuilleumier (2020) reported nonsignificant associations between the trait and the appraisals of Outcome Probability and Unpredictability. With regard to the appraisal of Causality/Agency, in Tong et al. (2006), no significant associations with its three declinations (Self, Others, and Circumstances) emerged. Similarly, in Mohammadi and Vuilleumier (2020) and Wilt (2014), Extraversion was not significantly related to Cause-chance and Agency-Self, respectively. In contrast, in Scherer (2020), Extraversion negatively predicted the appraisal of Other-cause. Regarding the appraisal of Certainty, two studies reported nonsignificant results (Tong et al., 2006; Wilt, 2014). Similarly, Sacchi and Dan-Glauser (2025) found that no appraisal belonging to the functional category of Implications was related to Extraversion.

#### Extraversion and Coping Potential

We found weak evidence for this association in the literature. While in Scherer et al. (2022) Extraversion was significantly and positively related to low Coping Potential, it was not the case for several other appraisals within this functional category (Sacchi & Dan-Glauser, 2025; Tong et al., 2006; Wilt, 2014).

#### Extraversion and Normative Significance

In Inglis et al. (2018), not only was the appraisal of Compatibility with Internal Standards positively correlated with Affiliative Extraversion, but it also significantly mediated the relationship between the trait and the emotional state of warmth-affection. Participants with higher Extraversion scores were also significantly less likely to appraise situations as unfair and violating moral standards (Tong et al., 2006). All these findings were however not supported by Mohammadi and Vuilleumier (2020), Sacchi and Dan-Glauser (2025), and Wilt (2014).

### Openness/Intellect and Cognitive Appraisals

#### Openness/Intellect and Relevance

Openness was positively and strongly correlated with the appraisal of Other-Unpleasantness (Mohammadi & Vuilleumier, 2020). Neither Subjective Unpleasantness (Mohammadi & Vuilleumier, 2020) nor Pleasantness (Sacchi & Dan-Glauser, 2025; Tong et al., 2006; Wilt, 2014) were however significantly related to this trait. Moreover, Openness did not show any significant association with the appraisals of Other-Relevance and Novelty (Mohammadi & Vuilleumier, 2020; Sacchi & Dan-Glauser, 2025).

#### Other Studies

In this section, we bring together different contributions that have investigated the appraisal processes leading to esthetic emotions, such as interest and pleasure, deriving from curious, explorative, and information-seeking behaviors (Silvia, 2005). In these studies, the appraisals of Relevance/Novelty and Coping Potential—found to be at the core of interest (Silvia, 2005, 2008; Silvia et al., 2009)—are revisited and expanded to include the more granular appraisals of Complexity, Uncertainty, Ambiguity, Contradiction, and Understandability. Moreover, a subset of these contributions endorses the personality variable of Trait Curiosity, conceptualized as a proxy for Openness (Silvia, 2008; Silvia et al., 2009).

Fayn, MacCann et al. (2015) explored the relationship between Openness/Intellect, measured with the Big Five Aspect Scale (DeYoung et al., 2007), and appraisal of Novelty/Complexity in the context of esthetic experience following the administration of 18 visual art stimuli. In the Big Five Aspect Scale, the trait comprises the two aspects of Openness and Intellect. The former reflects the artistic and unconventional side of the trait, related to the curious exploration of sensory patterns; the latter reflects erudition and intelligence, related to the curious exploration of abstract patterns (DeYoung et al., 2007). Both Openness and Intellect moderated the relationship between the appraisal of Novelty and the emotion of interest in a positive and strong direction. The relationship between the appraisal of Novelty and the emotion of pleasure was moderated solely by Openness, again in a positive and strong direction. In a subsequent study, Fayn et al. (2017) expanded their setup by presenting three distinct esthetic stimuli—artistic, scientific, and philosophical. The appraisal of Novelty did not mediate the relationship between Openness or Intellect and the emotion of Interest in any of the three stimulus categories. However, contrary to expectations, in the art stimulus condition, the appraisal of Novelty was significantly and negatively predicted by Openness. Moreover, further analyses revealed that Openness—but not Intellect—moderated the relationship between Novelty and interest in the context of science and philosophy stimuli. In other words, participants scoring higher on Openness not only responded to novel stimuli with greater interest, but also reported an increased experience of interest (Fayn et al., 2017).

#### Openness/Intellect and Implications

In Wilt (2014), Openness was positively correlated with the appraisals of Goal Conduciveness, Agency-Self, and Certainty. In contrast, in Tong et al. (2006) and Sacchi and Dan-Glauser (2025), Openness was not significantly related to these appraisals, nor to the appraisal of Agency-Circumstances in the former study. Openness was found to positively and significantly predict the appraisal of Agency-Others (Tong et al., 2006), and to strongly and negatively predict the appraisal of Chance-cause (Mohammadi & Vuilleumier, 2020). Finally, the trait did not emerge as a significant predictor of Unpredictability and Outcome Probability (Mohammadi & Vuilleumier, 2020; Sacchi & Dan-Glauser, 2025).

#### Openness/Intellect and Coping Potential

The trait did not significantly relate to any of the appraisals within this functional category (Sacchi & Dan-Glauser, 2025), including Control (Tong et al., 2006; Wilt, 2014) and Effort (Tong et al., 2006).

#### Other Studies

In an experiment showing highly complex esthetic stimuli, Silvia (2008) found that the appraisal of Coping Potential (“Understanding Potential”) fully mediated the relationship between Trait Curiosity and interest. In another experiment, where stimulus complexity and type were varied, trait Curiosity predicted the appraisal of Coping Potential for simple stimuli, with no mediation effect. However, for complex stimuli, appraisal of Coping Potential fully mediated the relationship between trait Curiosity and interest (Silvia, 2008). In the study by Fayn, Tiliopoulos et al. (2015), the appraisal of Understanding Potential mediated the relationship between Openness/Intellect and interest during the evaluation of quotes, but the effect was significant for Intellect only. In fact, the appraisal of Understanding Potential contributed less to the experience of interest in participants with higher levels of Openness. In contrast, in Fayn et al. (2017), the appraisal of Understanding Potential of visual art, science, and philosophy stimuli mediated the relationship between Openness and interest, while Intellect did not show such mediation.

Zajenkowski and Matthews (2019) employed a similar conceptualization of the appraisal of Understanding Potential, formulated as intelligence satisfaction and subjectively assessed intelligence. The authors found that only the trait aspect of Intellect correlated positively and significantly with these two revisited appraisals, while the aspect of Openness did not. Moreover, Intellect was associated with increased positive affect, hedonic tone and life satisfaction through greater satisfaction with one's intellectual capacities. Subjectively assessed intelligence mediated only the Intellect-life satisfaction relationship, in a positive direction (Zajenkowski & Matthews, 2019).

#### Openness/Intellect and Normative Significance

Multiple studies reported nonsignificant associations between the trait of Openness and Normative Significance appraisals (Mohammadi & Vuilleumier, 2020; Sacchi & Dan-Glauser, 2025; Tong et al., 2006; Wilt, 2014).

### Agreeableness and Cognitive Appraisals

#### Agreeableness and Relevance

In Mohammadi and Vuilleumier's (2020) study, a negative correlation between Agreeableness and the appraisal of Novelty emerged. In Scherer (2020), a composite variable of Conscientiousness and Agreeableness (“conscientious/agreeable”) positively and significantly predicted the appraisal of Relevance. In contrast, two studies reported no association with Personal-Relevance and Other-Relevance (Mohammadi & Vuilleumier, 2020; Sacchi & Dan-Glauser, 2025). Regarding the appraisal of Pleasantness, whereas in Scherer (2020) it was negatively predicted by the composite personality variable, a positive correlation emerged in Wilt (2014) when the Big-Five Inventory Agreeableness was considered. No significant associations were found, however, in Tong et al. (2006), Mohammadi and Vuilleumier (2020), and Sacchi & Dan-Glauser (2025).

#### Agreeableness and Implications

While in Wilt (2014), small positive correlations between the trait and the appraisal of Goal Conduciveness and Agency-Self emerged, no significant association was found in Tong et al. (2006). In addition, Agreeableness was weakly and negatively correlated with the appraisal of Unpredictability (Mohammadi & Vuilleumier, 2020). No significant associations were found with the other appraisal declinations of Agency/Cause (Mohammadi & Vuilleumier, 2020; Tong et al., 2006), or the appraisal of Certainty (Tong et al., 2006; Wilt, 2014). In Scherer's (2020) study, the “conscientious/agreeable” pooled variable negatively predicted the appraisal of Expectations, despite its small effect size (Scherer, 2020), while in Mohammadi and Vuilleumier (2020), no significant association was reported with the appraisal of Outcome Probability. In Sacchi and Dan-Glauser (2025), none of the appraisals within this functional category were related to Agreeableness.

#### Agreeableness and Coping Potential

In Wilt (2014), Agreeableness showed a slight positive association with the appraisals of Control, while no significant association was found with either Control or Effort in Tong et al. (2006). Again, none of the appraisals within this functional category were significantly associated with Agreeableness in Sacchi and Dan-Glauser (2025).

#### Agreeableness and Normative Significance

Three studies reported nonsignificant associations between the trait of Agreeableness and appraisals belonging to the Normative Significance category (Mohammadi & Vuilleumier, 2020; Tong et al., 2006; Wilt, 2014). In contrast, in the slightly ambiguous positive scenario of Sacchi and Dan-Glauser (2025), findings indicated that the appraisal of External Standards Incompatibility marginally and positively mediated the relationship between Agreeableness and both the intensity and duration of the emotional episode.

### Conscientiousness and Cognitive Appraisals

#### Conscientiousness and Relevance

In Tong et al. (2006), participants with higher Conscientiousness scores were more likely to appraise events as pleasant for themselves, while in Scherer (2020), the “conscientious/agreeable” pooled variable predicted negatively the appraisal of Valence (i.e., Pleasantness). A similar pattern to the former emerged in Wilt (2014), although the result did not reach statistical significance. Mohammadi and Vuilleumier (2020) reported no significant association with the appraisal of Subjective Unpleasantness, but a small positive one with Other-Unpleasantness was observed. Regarding the appraisal of Relevance, Scherer (2020) found a positive association with the composite trait. In contrast, no significant association with the Other-Relevance appraisal was found in Mohammadi and Vuilleumier (2020), nor with that of Novelty. None of the appraisals within this category were significantly associated with Conscientiousness in Sacchi and Dan-Glauser (2025).

#### Other Studies

Conscientiousness was significantly and positively correlated with the appraisal of Pleasantness for academic-approach and performance-evaluative stimuli (Cummings et al., 2019), but not of academic-avoidance ones. Similarly, the trait was significantly correlated with the appraisal of Relevance/Significance across these stimuli as well as academic-avoidance ones, though in a negative direction.

#### Conscientiousness and Implications

Tong et al. (2006) reported that conscientious participants were more likely to appraise events as certain, a finding not replicated in Wilt (2014). Wilt (2014) reported a significant positive association between Conscientiousness and Goal Conduciveness. Tong et al. (2006) observed a similar but nonsignificant pattern. Regarding the appraisal of Agency/Cause, in Mohammadi and Vuilleumier (2020), Conscientiousness strongly and negatively correlated with the appraisal of Chance-cause. Again, Tong et al. (2006) reported a similar but nonsignificant result. Moreover, their findings indicated nonsignificant and significantly negative associations with the appraisals of Agency-Self and Agency-Others, respectively. In contrast, Wilt (2014) found a positive correlation between Conscientiousness and the appraisal of Agency-self. In Mohammadi and Vuilleumier (2020), Conscientiousness was positively correlated with the appraisal of Unpredictability, while no significant association was reported for the appraisal of Outcome Probability. In Scherer (2020), the composite trait variable negatively correlated with the appraisal of Expectations, although the effect size was small (Scherer, 2020). Again, none of the appraisals within this category were significantly associated with Conscientiousness in Sacchi and Dan-Glauser (2025).

#### Conscientiousness and Coping Potential

Participants higher in Conscientiousness were more likely to appraise events as within their control (“Perceived Control”; Tong et al., 2006; Wilt, 2014). However, Conscientiousness was unrelated to Effort (Tong et al., 2006). Notably, Sacchi and Dan-Glauser (2025) found that the appraisal of Powerlessness negatively mediated the relationship between Conscientiousness and both the experiential and physiological components of the emotional episode, but only in their positive scenario.

#### Conscientiousness and Normative Significance

Participants scoring higher on Conscientiousness were significantly less likely to appraise situations as unfair and violating moral standards (Tong et al., 2006). In contrast, three studies reported nonsignificant associations between the trait and appraisals within the category of Normative Significance (Mohammadi & Vuilleumier, 2020; Sacchi & Dan-Glauser, 2025; Wilt, 2014).

## Discussion

The goals of this narrative review were twofold. The first was to summarize the evidence on the associations between personality traits and cognitive appraisal, drawing from the CPM as the most comprehensive structural model among modern appraisal theories. To that end, we situated these findings within the integrative model of the Big Five trait taxonomy (John & Srivastava, 1999; McCrae, 2020), following the recommendations of Bainbridge et al. (2022).

The second goal was to provide an explanatory perspective on these associations. In a recent summary of the limited extant knowledge on the topic, Reisenzein et al. (2020) identified three processes plausibly responsible for interindividual differences in appraisal at the information-processing level: (a) stable differences in *beliefs* and *desires*—here, we specifically focus on desires, as beliefs, conceptualized as stable dispositional traits, substantially overlap with the Big Five; see Bainbridge et al. (2022); Baumert et al. (2019); Poluektova et al. (2023); (b) *chronic accessibility* of appraisal-relevant structures, defined as the individual's readiness to recognize certain stimuli (Robinson, 2007) paired in memory with valenced evaluations, and (c) differences in *habitual processing* procedures, defined as the mode through which these stimuli are attended via attention deployment (Matthews, 2020). Given that Reisenzein et al. (2020) primarily emphasize beliefs and desires (i.e., essential basic goals and needs), we here complement their work by expanding on the other two processes.

### Desires, Chronic Accessibility, and Habitual Processing Relate to Appraisal

Historically, in appraisal research, these three mechanisms were already proposed as crystalized and automatic modalities (as opposed to effortful ones) through which appraisal could be quickly elicited (Reisenzein, 2001; Smith & Kirby, 2001). This automatic, rapid cognitive processing is part of a hardwired and evolutionary system conserved across species, which relies on learned “lessons” and preferences (*habitual processing*), retrieved from memory (*accessibility*) to foster goal-directed (*desired*) emotions and behaviors necessary for survival (Ellsworth & Scherer, 2003). Hence, when faced with a novel situation, an organism needs to quickly orient toward and prepare for unexpected, unpleasant, and potentially dangerous situational features, which could then be escaped and avoided in the future to secure survival and wellbeing (Ellsworth & Scherer, 2003). This explains why the appraisals of Novelty, Pleasantness, and Goal Relevance are believed to be among the most automatically endorsed forms of appraisal (Ellsworth & Scherer, 2003).

### Desires, Chronic Accessibility, and Habitual Processing Relate to Personality

In addition to clarifying the personality–appraisal relationship through the three mentioned processes, Reisenzein et al. (2020) speculate about their status as personality determinants of appraisal. Concerning *chronic accessibility* and *habitual processing*, this status is supported by evidence from experimental approaches to personality (Robinson, 2021; Robinson & Irvin, 2022; Robinson et al., 2023). For instance, Neuroticism is associated with strong negative memory networks, while Agreeableness with enhanced self-regulatory capacities when processing hostile cues (Robinson, 2021). These mechanisms might constitute the cognitive processes through which the Big Five personality traits influence proximal outcomes, such as negative and positive affects (Robinson, 2021), and possibly also distal outcomes, such as life satisfaction or health (Keltner & Shiota, 2021). Concerning *desires*, the concept of approach and avoidance as motivational drives underlying behavior has gained widespread acceptance in both emotion (Elliot et al., 2013) and personality research (Corr et al., 2013).

This is especially true for behaviors typically captured by the Big Five traits (Corr & Krupić, 2020; Robinson, 2021). The Big Five literature has established that Neuroticism closely tracks punishment sensitivity and reactivity (Keiser & Ross, 2011; Robinson, 2021; Smits & Boeck, 2006), which results in avoidance motivation and passive avoidant behavior as emotional coping strategies (Corr & Krupić, 2017; Sauer-Zavala et al., 2021; Semcho et al., 2023). In contrast, Extraversion closely tracks reward sensitivity and reactivity, and approach motivation (Keiser & Ross, 2011; Robinson, 2021; Smits & Boeck, 2006). Similarly, Openness/Intellect relies on cognitive forms of approach to stimuli perceived as rewarding, fueled by curiosity and exploration (Corr et al., 2013). Finally, the motivational bases of Agreeableness and Conscientiousness appear to be more complex, involving interactions between reward approach (e.g., completing a task and helping others) and punishment avoidance behaviors (e.g., being unreliable and letting down others) as hypothesized by Corr et al. (2013). Motivational accounts of the Big Five are theorized to be biologically anchored, operating through substrates that can explain different forms of behavior (Corr et al., 2013; DeYoung, 2015).

### The Three Processes Involvement in the Trait–Appraisal Relationship

Based on the gathered evidence, we next examine the results trait-by-trait, taking the integrative and three-process explanatory stances as our guiding principles. We recognize that the three mechanisms identified by Reisenzein et al. (2020) might not fully explain the observed relationships between personality and appraisal. Nonetheless, we believe that these mechanisms represent a pivotal starting point for clarifying how traits and appraisals are interconnected.

#### Neuroticism

Overall, regardless of the nature of the stimuli, Neuroticism appears to be related to more negative types of appraisals and to the experience of negative emotions, marking a rigid and vulnerable character. As shown in Table 2, the appraisals of Pleasantness, Predictability, Agency, Control and Fairness are the most relevant to the trait of Neuroticism, all showing negative associations.

The findings for the appraisals of Pleasantness and Predictability can be contextualized in the well-established presence of an *information processing* (i.e., attentional) and *accessibility* (i.e., memory) bias toward negative stimuli in individuals higher in Neuroticism. Not only do they attend to negative information rather than diverting their attention away, but also, given their intense use of dysfunctional coping strategies and styles such as rumination and perseveration, their negative thoughts are more interconnected and more quickly accessible in memory than positive ones (Robinson, 2021). Concerning *desires*, those scoring higher on Neuroticism show increased sensitivity to punishment-related feedback, as evidenced by their tendency to frequently revise their choices after incorrect responses in a categorization task (Robinson, 2021). Moreover, they exhibit a pronounced tendency toward perceptual avoidance, operationalized as a tendency to endorse greater distance between the self and the stimuli (Robinson et al., 2013). This mechanism also helps explain the negative association found with the appraisal of (lack of) Agency, given that passive behavioral avoidance is a well-known correlate of Neuroticism (Corr & Krupić, 2017).

Arguably, among those scoring higher on Neuroticism, passive avoidance contributes to diminished perceptions of control, particularly in situations already interpreted as negative. Indeed, such individuals appraise stressful events as more threatening and less challenging than their counterparts (Kilby et al., 2018). Moreover, they appraise situational demands as very high, and their coping ability as very low (Tomaka & Magoc, 2021). Additional evidence from methodologically diverse studies converges on lower self-esteem and self-efficacy as common correlates of the trait (Judge et al., 2002; Mu et al., 2019; Robinson & Meier, 2005; Zeigler-Hill et al., 2015). Individuals higher in Neuroticism also report more frequent daily hassles and stronger emotional reactivity directed towards them, compared to those scoring lower on the trait (Suls & Martin, 2005). These findings align with results from the two differently valenced daily-life scenarios in Sacchi and Dan-Glauser (2025), and from the ecological momentary assessment studies by Tong (2010) and Tong et al. (2006), but not from Wilt (2014). This latter discrepancy may stem from differences in sample characteristics: while participants in Wilt (2014) belong to the community, participants in Tong et al. (2006) were police officers. It is plausible that police officers encounter more negative situations than community dwellers. The discrepancy could also lie in the sample size differences, with Wilt (2014) having one quarter of the participants of Tong (2010).

Finally, regarding the appraisal of Fairness, given the darker interpretive lens through which individuals higher in Neuroticism perceive and process the world (Uziel, 2006), it is not surprising that they report higher victim sensitivity—the perception of being the potential victim of injustice—and react more strongly to it (Schmitt et al., 2010). Justice sensitivity appears to be more strongly linked to Neuroticism as a form of self-focused, rather than other-focused, concern (Baumert et al., 2014), potentially grounded in motivational processes.

#### Extraversion

Overall, Extraversion appears to be related to more positive types of appraisals, and to the experience of positive emotions. As shown in Table 2, the appraisals of Relevance, Pleasantness, Goal Conduciveness, and Internal/External Standards Compatibility emerged as the most relevant to the trait of Extraversion. However, the anticipated strong link with appraisals within the Coping Potential category was not supported.

The findings concerning the Relevance appraisal can be contextualized in the well-documented experimental association between Extraversion and an *attentional bias* toward positive (rewarding) stimuli, a link that appears to further promote cognitive approach to such cues (Derryberry & Reed, 1994; Robinson, 2021). For example, in one study by Amin et al. (2004), extraverts were found to shift their attention away from negative stimuli during exposure to negative/neutral pictures. Given the strong link between Extraversion and Positive Affect (Wilt & Revelle, 2017), Extraversion should lead not only to avoidance of negative stimuli, but also to maximized attraction to rewarding stimuli (Corr et al., 2013; Robinson et al., 2013). Indeed, with an affective priming task, it has been shown that when individuals higher in Extraversion identify a positive word, the recognition of a subsequent positive one is significantly faster compared to their counterparts. Robinson (2021) explains this phenomenon as a cognitive manifestation of reward reactivity. Interestingly, in a recent microlongitudinal study on retrospective memory reporting, highly extraverted/low neurotic individuals were found to report exaggerated high arousal of positive affect and to underreport low arousal of positive affect immediately following the end of the 10-day study (Lay et al., 2017). These individuals may thus attend more closely to rewarding cues, which could enhance their emotional responses and increase memory accessibility for such stimuli.

The relationship between Extraversion and the appraisal of Pleasantness appears more nuanced than expected. For example, while the observed positive relations align with the social and reward-seeking nature of extraverts (Wilt & Revelle, 2017), a negative correlation with Unpleasantness for others (“Feel it was unpleasant for someone else”; Mohammadi & Vuilleumier, 2020) also emerged. This suggests that highly extraverted individuals may not fully recognize the discomfort others experience in a given situation, revealing a discrepancy with respect to what we know about extraverts. Indeed, it is well-established that extraverts are energetic, assertive, dominant, and strongly socially oriented, manifested even in their implicit tendency to pair “people”-related words with “reward” (Wilkowski & Ferguson, 2014). However, despite these strong interpersonal skills, they are not perceived as good listeners, as shown by Flynn et al. (2023). Supporting this view, in their comprehensive study, Melchers et al. (2016) found that two subfacets of Empathy—that is, Perspective Taking, the cognitive aspect of Empathy, and Fantasy, described as the “Ability to transpose themselves into feelings, thoughts and actions of fictional characters” (Melchers et al., 2016, p. 3)—were only weakly or nonsignificantly related to Extraversion, with small effect sizes. A lack of awareness of others’ discomfort, if pronounced, could potentially reinforce socially inappropriate behaviors: indeed, a recent meta-analysis found that a personality profile characterized by high Extraversion and Neuroticism, and low Agreeableness and Conscientiousness, was associated with bullying and victimization (Mitsopoulou & Giovazolias, 2015).

The positive relationship between Extraversion and the appraisal of Goal Conduciveness is consistent with the motivational nature of this appraisal (Ellsworth & Scherer, 2003), and the approach motivation (*desire*-driven) signature of the trait, that is, oriented toward the agentic pursuit of goals and their attainment (Corr & Krupić, 2020). Supporting this view, Wilt et al. (2017) found that the construct of approach goal was positively predicting state Extraversion, which in turn influenced state Positive Affect through the mediating role of Velocity—operationalized as the perceived progress toward a goal. Behavioral manifestations of Extraversion, such as talkativeness, assertiveness, and cheerfulness, may thus be linked to goal pursuit via this dynamic feedback loop (Wilt et al., 2017). This affective “virtuous cycle” may also account for the increased frequency of positive life events reported by extraverts in personal, social, and occupational domains (Wilt & Revelle, 2017). Similarly, it may explain the pattern of findings related to Normative Significance: extraverts may shape their environments in ways that reduce encounters with unfairness or injustice.

#### Openness/Intellect

Overall, Openness/Intellect appears to be related to more positive types of appraisals. As shown in Table 2, the appraisals of Novelty, Agency, and Understanding Potential emerged as the most relevant to the trait of Openness.

Given the curious and explorative (*desire*-driven) nature of the trait (Corr et al., 2013; Sutin, 2017), we hypothesized a positive relation with the appraisal of Novelty, which was supported by the data. Attentional processes seem also to be implicated. Individuals higher in Openness exhibit attenuated latent inhibition, a survival-based mechanism through which nonreinforced stimuli are considered irrelevant and discarded from attention (Peterson & Carson, 2000; Peterson et al., 2002). This means that highly open individuals do not disengage from familiar stimuli even after repeated exposure, which confers them a performance advantage in rule decoding tasks (Peterson & Carson, 2000; Peterson et al., 2002). Those higher in Openness thus find information intrinsically rewarding, exhibiting a maximized approach-oriented response to novelty (Corr et al., 2013).

Results were more nuanced when distinguishing between the two aspects of Openness and Intellect. While the former appears to explain the excitement and pleasantness experienced when confronted with artistic stimuli, the latter is more strongly related to interest in highly complex stimuli, consistent with a high need for cognition and cognitive challenge (Sutin, 2017). Indeed, higher Intellect, rather than Openness, has recently been found to drive the experience of mixed percept, a form of inclusive perceptual experience in which attention toward two competing visual stimuli is not divided, but instead, is channeled to integrate the rivalry stimuli (Antinori et al., 2017). This suggests a flexible cognitive mechanism for engaging with visual complexity. Given this effortless *information-processing* of complexity, it is not surprising that Openness/Intellect is linked to a greater sense of mastery and autonomy, contextualizing the findings regarding the Agency and Understanding Potential appraisals. This may be due to the endorsement of optimal self-regulation and adaptive coping strategies (Sutin, 2017) such as engagement coping, a proactive coping style that includes support-seeking, cognitive restructuring, and acceptance, and that mitigates the emotional consequences of stress (Carver & Connor-Smith, 2010). Indeed, open/intellectual individuals do perceive themselves as able to cope with stressful situations, instead of seeing these stressors as less demanding, as extraverts do (Tomaka & Magoc, 2021).

#### Agreeableness

As shown in Table 2, the findings for the trait of Agreeableness appear sparser than those for the other traits, with the appraisals of Relevance, Pleasantness, and Predictability potentially being the most relevant to the trait. These associations can be contextualized within experimental work on the implicit cognitive self-regulatory abilities at higher levels of Agreeableness (Robinson, 2021), tapping onto positive *chronic accessibility* and *habitual processing* mechanisms (Reisenzein et al., 2020). Indeed, it appears that highly agreeable individuals are able not only to control hostile thoughts by “blocking” blame accessibility, but also, to more rapidly recruit prosocial cognitions after being exposed to hostile primes (Robinson, 2021). Moreover, Bresin and Robinson (2015) found that the emotion regulation strategy of situation selection—highly effective in regulating negative emotions and promoting positive ones—is a notable correlate of Agreeableness. Arguably, situation selection also impacts the types of appraisals that are likely to unfold following the unraveling of the chosen situation: by constraining the environment to more familiar or comfortable situations, the likelihood of experiencing something completely new or unpredictable is reduced, while the chances of goal achievement (Goal Conduciveness) are higher. In Mohammadi and Vuilleumier (2020), participants viewed video clips, which clearly limited their agency over the environment. However, given the empathic, warm, and altruistic predisposition of agreeable individuals (Graziano & Tobin, 2017), they may have been more attuned to the unfolding events, rendering the situations less novel and more predictable.

The results for the Relevance appraisal, and in particular the discrepant ones regarding the Pleasantness appraisal, can be attributed to the different methodologies employed, and may be better understood through the motivational (*desires*) foundation of Agreeableness (Corr et al., 2013). In Scherer's (2020) study, participants were presented with six negative real-life scenarios: being late to work, losing a job, forgetting an important appointment, experiencing relationship difficulties, overhearing a friend speaking badly about them, and dealing with a cheating partner. The appraisal ratings were then pooled across these scenarios to obtain an overall appraisal score for each participant. Given the personal investment that agreeable individuals make in affiliative relationships, situations involving betrayal or conflict with close others are both unpleasant and personally relevant. In contrast, Wilt’s (2014) results align with evidence linking Agreeableness to lower intensity of negative emotion experiences, greater life satisfaction, and better interpersonal functioning, possibly achieved through situation selection (Bresin & Robinson, 2015; Graziano & Tobin, 2017). Interestingly, Scherer’s (2020) results mirror those of Suls et al. (1998), who found that agreeable individuals, when faced with negative interpersonal conflicts, were found to react with more stress—underscoring that their affiliative orientation may make relational threats particularly impactful.

#### Conscientiousness

As shown in Table 2, the appraisals of Relevance, Pleasantness and Agency emerged as the most relevant to the trait of Conscientiousness. However, the expected strong associations with the functional categories of Coping Potential and Normative Significance were not confirmed.

Even though the emotional nature of Conscientiousness remains partially unclear (Reisenzein et al., 2020), a motivational (*desire*-based) perspective may help explain some of its affective features. While Conscientiousness could appear as being exclusively a reward-approach trait—evidenced by its achievement striving tendency and relation to important life outcomes, such as academic performance (Noftle & Robins, 2007), career achievements and satisfaction (Jackson & Roberts, 2017), as well as success (Trapmann et al., 2007)—some accounts also suggest it entails a component of punishment avoidance. For instance, acting conscientiously to prevent failure or moral transgression may result in occasional anxiety (Corr et al., 2013). In this regard, guilt proneness, defined as the anticipation of guilt and efforts to avoid it, has emerged as one of the few consistent emotional correlates of the trait (Fayard et al., 2012).

In the reviewed literature, the appraisals of Relevance and Pleasantness were often tested in the context of stimuli that were particularly motivationally salient to Conscientiousness, such as positive academic-stimuli (Cummings et al., 2019), or negative real-life scenarios, depicting work and social-related misbehaviors (Scherer, 2020). In this latter study, we can speculate that the negative instances are utterly inconsistent with the diligent and detailed-oriented attitude of conscientious individuals, leading to appraisals of high Relevance and low Pleasantness. The positive associations reported in the ecological momentary assessment studies of Tong et al. (2006) and Wilt (2014) may also reflect context effects. Indeed, one cannot exclude the possibility of conscientious individuals being more likely to encounter or select environments consistent with their goals and values, which they subsequently appraise as more pleasant. For example, the daily behavior most associated with Conscientiousness was found to be studying (Wilt & Revelle, 2019), an activity that conscientious people likely find pleasant and plausibly consistent with an approach motivation account of the trait (Corr et al., 2013). However, it should be noted that, in Scherer (2020), the use of pooled Agreeableness-Conscientiousness scores might have obscured distinctions between the two traits, yielding “omnibus” effects that are harder to interpret. In contrast, Mohammadi and Vuilleumier (2020) found that Conscientiousness was marginally related to a more objective and detached situational evaluation of Others-Unpleasantness. In other words, those higher in Conscientiousness appeared to take the other person's viewpoint by objectively rating a video clip as slightly more unpleasant for someone else. This aligns with findings from Melchers et al. (2016), in which the trait's link to empathy was driven primarily by the Perspective Taking component.

Emerging evidence also points to specific neurobiological substrates underlying information processing in Conscientiousness (DeYoung & Blain, 2020). Certain unique features, such as impulse control, planning, and distraction avoidance, are associated with prefrontal cortex activity, a brain region located in what is known as the salience/ventral attention network (DeYoung & Blain, 2020). Recent evidence has confirmed this network as a key substrate of Conscientiousness (Sassenberg et al., 2023). Because this substrate underlies goal prioritization and attentional redirection toward goal-relevant cues, it likely facilitates the *habitual processing* of these trait-salient stimuli, while discarding nonrelevant ones. This evidence could explain further our descriptive associations between the trait and the subordinate appraisal checks of Expectations and Goal Relevance.

Finally, regarding the appraisal of Agency, Conscientiousness is positively related to a higher internal locus of control (Nudelman & Otto, 2021), which could explain why highly conscientious individuals appraise stressful situations as more controllable (Kilby et al., 2018). This provides additional support for the trait's positive association with the appraisal of Control and its negative association with Powerlessness.

#### A Brief Summary of the Results Under an Integrative Process-Based Framework

Overall, at the descriptive level, the relationship between Neuroticism and Extraversion on the one hand and cognitive appraisals on the other hand aligns with the wealth of existing experimental and observational literature on the traits preponderant affective nature and sequalae (Revelle & Scherer, 2009). Concerning the other traits, we think that a new perspective emerges, since these have not been consistently investigated in the emotion field. The focus on Openness, Agreeableness and Conscientiousness, and their relationship to appraisal processes, has thus sharpened the current understanding of their differential implications in the emotional sphere.

At the explanatory level, the three mechanisms of *desires*, *accessibility*, and *information processing* effectively account for the ways in which Neuroticism and Extraversion, and to a lesser extent Agreeableness, related to appraisals. For the remaining traits, a more differentiated pattern emerge. Specifically, Openness and Conscientiousness appear to operate primarily through the mechanisms of *information processing* and *desires*. Even for these less researched traits in the emotion field, the three processes offer an additional layer of understanding that is indeed needed in this intersectional field (Poluektova et al., 2023). Overall, we believe that the three processes act as a conceptual bridge between personality and appraisal, forming what we could refer to as a personality-appraisal process-based framework.

## Limitations and Future Directions

The present narrative review presents several limitations. The first concerns the heterogeneity in samples, methodological approaches and stimuli across the reviewed studies, which likely contributed to inconsistencies in the results and may have also led to the nonsignificant findings observed. When results were absent, this may have reflected a genuine lack of effect, but also the challenge of capturing an abstract mental phenomenon like cognitive appraisal—particularly via questionnaires. Social desirability issues or awareness difficulties over these semi-automatic processes may have further biased responses. There is a pressing need for more collaborative studies that integrate descriptive, laboratory, and daily-life assessments using a multicomponential framework (Scherer & Moors, 2019). This would allow for the systematic investigation of state and trait personality, appraisal processes, and emotional responses within a coherent line of research.

The second limitation relates to the scope of this review. It addresses a highly specific intersection between emotions and personality but, despite its effort to be as comprehensive as possible, some studies may have been overlooked. Nonetheless, we argue that this review was both timely and necessary, given the paucity of systematic syntheses in this domain (Reisenzein et al., 2020). Indeed, given its explanatory and integrative nature, our review can serve as a blueprint for other researchers to navigate the intersectional maze of personality and affective research.

The third limitation concerns the sole endorsement of the Big Five trait model. We acknowledge the existence of alternative models of personality that extend beyond five dimensions, including both normative frameworks—such as the six-factor HEXACO model (Lee & Ashton, 2004) and maladaptive ones, such as the Dark Triad model (Paulhus & Williams, 2002). Because appraisal research has seldom endorsed alternative personality models, we did not incorporate these in the present work. Of note, in the HEXACO model, item content is redistributed differently across traits than in the Big Five: HEXACO Emotionality (i.e., the inverse of Neuroticism) lends anger-related items to HEXACO Agreeableness, while borrowing empathy-related items from Big Five Agreeableness (Ludeke et al., 2019). Moreover, HEXACO Agreeableness appears to share variance with Extraversion and Emotionality. The model also introduces a sixth dimension of Honesty-Humility, which comprises non-self-serving and moral tendencies (Ludeke et al., 2019). Therefore, future studies could examine whether diverse trait–appraisal associations emerge when endorsing the HEXACO framework.

Before concluding, we propose two further avenues to refine understanding in personality–emotion research. First, motivational (*desire*) forces appear to be a transversal mechanism across traits. Thus, future studies might adopt the biologically grounded meta-traits of Stability and Plasticity (DeYoung, 2015; Digman, 1997) as a higher, overarching explanatory framework for trait–appraisal associations, and extend it to more distal emotional outcomes. Stability, comprising the traits of Emotional Stability, Agreeableness and Conscientiousness, serves a conservative and restraining function by protecting individuals’ goals from emotional, social, and motivational disruptions; Plasticity, comprising Extraversion and Openness, serves an approach and exploratory function by enabling cognitive and behavioral engagement (DeYoung, 2015). In appraisal research, recent neuropsychological evidence has identified coordinated neural activity underlying qualitatively different types of appraisals and emotions generation (Mohammadi et al., 2023). The related brain networks may overlap with those identified for Stability and Plasticity (DeYoung & Blain, 2020), possibly via serotonergic and dopaminergic pathways. More experimental work is needed to clarify their plausible synergistic interaction.

Second, we emphasize that many studies reviewed here employed moderation and mediation models to explore the personality–appraisal–emotion relationship (see last column of Table 1). Moderation implies that traits alter the strength or direction of the appraisal–emotion link, thereby providing an explanation for the high heterogeneity of emotional experience (Hampson, 2021). Mediation implies that appraisal constitutes the causal mechanism through which personality exerts its effects on emotional (Sacchi & Dan-Glauser, 2025) and possibly social and life outcomes (Keltner & Shiota, 2021). Further exploration of both approaches is needed, as well as more research on personality and other emotion components (such as physiology, action tendencies, and expressivity) under a multicomponential lens—although establishing causal relationships remains complex (Scherer & Moors, 2019). While *chronic accessibility*, *habitual processing*, and *desires* have been proposed as personality antecedents of appraisal (Reisenzein et al., 2020), it remains to be determined whether these biases and motivational drives are part of, reside at the level of, or are influenced by personality.

## Conclusion

To conclude, by expanding on the three overarching mechanisms of *desires*, *chronic accessibility*, and *habitual processing* (Reisenzein et al., 2020), and by adopting the well-established Big Five personality taxonomy as our organizing framework (Bainbridge et al., 2022), we have provided an explanatory and integrative perspective on the evidence accrued on the personality–appraisal relationship. This approach refines our understanding of how individual traits differentially influence emotional processes, through mechanisms that may be amenable to change and therefore represent potential targets for intervention (Sauer-Zavala et al., 2021). The prospect of targeting these malleable mechanisms offers a promising direction for future research, one that could further elucidate the affective sequelae and psychological complexity of personality.

## Supplemental Material

sj-docx-1-emr-10.1177_17540739251372161 - Supplemental material for Understanding the Relationship Between the Big Five Personality Traits and the Cognitive Appraisals Leading to Emotions: An Integrative Narrative Review Supplemental material, sj-docx-1-emr-10.1177_17540739251372161 for Understanding the Relationship Between the Big Five Personality Traits and the Cognitive Appraisals Leading to Emotions: An Integrative Narrative Review by Livia Sacchi and Elise Dan-Glauser in Emotion Review
