# Supplementary material for: Understanding the Relationship Between the Big Five Personality Traits and the Cognitive Appraisals Leading to Emotions: An Integrative Narrative Review
Source: Emot Rev. 2025 Sep 26;18(1):15–41. doi: 10.1177/17540739251372161 (PMC12758648; doi:10.1177/17540739251372161)
Supplement: sj-docx-1-emr-10.1177_17540739251372161 - Supplemental material for Understanding the Relationship Between the Big Five Personality Traits and the Cognitive Appraisals Leading to Emotions: An Integrative Narrative Review [file sj-docx-1-emr-10.1177_17540739251372161.docx]

**Appendix**

Search Terms for “Understanding the Relationship between the Big Five Personality Traits and the Cognitive Appraisals Leading to Emotions: An Integrative Narrative Review”

**# PsycNet (APA)**

1. cognitive appraisal, big five
2. big five, component process model
3. component process model, personality
4. Individual differences AND Keywords: appraisal AND Peer Reviewed Journals only
5. appraisal relevance, Neuroticism
6. appraisal novelty, Neuroticism
7. appraisal pleasantness, Neuroticism
8. appraisal goal relevance, Neuroticism
9. appraisal implications, Neuroticism
10. appraisal motive, Neuroticism
11. appraisal causality, Neuroticism
12. appraisal outcome probability, Neuroticism
13. appraisal discrepancy, Neuroticism
14. appraisal goal conduciveness, Neuroticism
15. appraisal urgency, Neuroticism
16. appraisal Coping Potential, Neuroticism
17. appraisal control, Neuroticism
18. appraisal power, Neuroticism
19. appraisal adjustment, Neuroticism
20. appraisal Normative Significance, Neuroticism/ appraisal internal standard compatibility, Neuroticism/ appraisal external standard compatibility, Neuroticism/ appraisal moral values, Neuroticism
21. appraisal relevance, Extraversion
22. appraisal novelty, Extraversion
23. appraisal pleasantness, Extraversion
24. appraisal goal relevance, Extraversion
25. appraisal implications, Extraversion
26. appraisal motive, Extraversion
27. appraisal causality, Extraversion
28. appraisal outcome probability, Extraversion
29. appraisal discrepancy, Extraversion
30. appraisal goal conduciveness, Extraversion
31. appraisal urgency, Extraversion
32. appraisal Coping Potential, Extraversion
33. appraisal control, Extraversion
34. appraisal power, Extraversion
35. appraisal adjustment, Extraversion
36. appraisal Normative Significance, Extraversion /appraisal internal standard compatibility, Extraversion/appraisal external standard compatibility, Extraversion/ appraisal moral values, Extraversion
37. appraisal relevance, Openness
38. appraisal novelty, Openness
39. appraisal pleasantness, Openness
40. appraisal goal relevance, Openness
41. appraisal implications, Openness
42. appraisal motive, Openness
43. appraisal causality, Openness
44. appraisal outcome probability, Openness
45. appraisal discrepancy, Openness
46. appraisal goal conduciveness, Openness
47. appraisal urgency, Openness
48. appraisal Coping Potential, Openness
49. appraisal control, Openness
50. appraisal power, Openness
51. appraisal adjustment, Openness
52. appraisal Normative Significance, Openness/ appraisal internal standard compatibility, Openness/ appraisal external standard compatibility, Openness / appraisal moral values, Openness.
53. appraisal relevance, Agreeableness
54. appraisal novelty, Agreeableness
55. appraisal pleasantness, Agreeableness
56. appraisal goal relevance, Agreeableness
57. appraisal implications, Agreeableness
58. appraisal motive, Agreeableness
59. appraisal causality, Agreeableness
60. appraisal outcome probability, Agreeableness
61. appraisal discrepancy, Agreeableness
62. appraisal goal conduciveness, Agreeableness
63. appraisal urgency, Agreeableness
64. appraisal Coping Potential, Agreeableness
65. appraisal control, Agreeableness
66. appraisal power, Agreeableness
67. appraisal adjustment, Agreeableness
68. appraisal Normative Significance, Agreeableness/ appraisal internal standard compatibility, Agreeableness / appraisal external standard compatibility, Agreeableness/ appraisal moral values, Agreeableness.
69. appraisal relevance, Conscientiousness
70. appraisal novelty, Conscientiousness
71. appraisal pleasantness, Conscientiousness
72. appraisal goal relevance, Conscientiousness
73. appraisal implications, Conscientiousness
74. appraisal motive, Conscientiousness
75. appraisal causality, Conscientiousness
76. appraisal outcome probability, Conscientiousness
77. appraisal discrepancy, Conscientiousness
78. appraisal goal conduciveness, Conscientiousness
79. appraisal urgency, Conscientiousness
80. appraisal Coping Potential, Conscientiousness
81. appraisal control, Conscientiousness
82. appraisal power, Conscientiousness
83. appraisal adjustment, Conscientiousness
84. Appraisal Normative Significance, Conscientiousness / appraisal internal standard compatibility, Conscientiousness / appraisal external standard compatibility, Conscientiousness / appraisal moral values, Conscientiousness

**# ISI Web of Knowledge**

1. cognitive appraisal AND big five
2. appraisal AND big five
3. (big five) AND TOPIC: (component process model)
4. TOPIC: (component process model) AND TITLE: (personality)
5. TITLE:(individual differences) AND TITLE:(appraisal)
6. appraisal AND Neuroticism
7. appraisal AND Extraversion
8. appraisal AND Openness
9. appraisal AND Conscientiousness
10. appraisal AND Agreeableness
11. TOPIC:(appraisal relevance) AND TOPIC:(Neuroticism)
12. TOPIC:(appraisal novelty) AND TOPIC:(Neuroticism)
13. TOPIC:(appraisal pleasantness) AND TOPIC:(Neuroticism)
14. TOPIC:(appraisal goal) AND TOPIC:(Neuroticism)
15. TOPIC:(appraisal aims) AND TOPIC:(Neuroticism)
16. TOPIC:(appraisal implications) AND TOPIC:(Neuroticism)
17. TOPIC:(appraisal motive) AND TOPIC:(Neuroticism)
18. TOPIC:(appraisal agency)  AND TOPIC:(Neuroticism)
19. TOPIC:(appraisal consequences)  AND TOPIC:(Neuroticism)
20. TOPIC:(appraisal causality)  AND TOPIC:(Neuroticism)
21. TOPIC:(appraisal outcome probability)  AND TOPIC:(Neuroticism)
22. TOPIC:(appraisal goal conduciveness)  AND TOPIC:(Neuroticism)
23. TOPIC:(appraisal urgency) AND TOPIC:(Neuroticism)
24. TOPIC:(appraisal expectations) AND TOPIC:(Neuroticism)
25. TOPIC: (appraisal coping potential) AND TOPIC: (Neuroticism)
26. TOPIC:(appraisal control) AND TOPIC:(Neuroticism)
27. TOPIC:(appraisal power) AND TOPIC:(Neuroticism)
28. TOPIC:(appraisal dominance) AND TOPIC:(Neuroticism)
29. TOPIC:(appraisal adjustment) AND TOPIC:(Neuroticism)
30. TOPIC:(appraisal normative significance) AND TOPIC:(Neuroticism)
31. TOPIC:(appraisal internal standard compatibility) AND TOPIC:(Neuroticism)
32. TOPIC:(appraisal external standard compatibility) AND TOPIC:(Neuroticism)
33. TOPIC:(appraisal norms) AND TOPIC:(Neuroticism)
34. TOPIC:(appraisal values) AND TOPIC:(Neuroticism)
35. TOPIC:(appraisal moral values) AND TOPIC:(Neuroticism)
36. TOPIC:(appraisal relevance) AND TOPIC:(Extraversion)
37. TOPIC:(appraisal novelty) AND TOPIC:( Extraversion)
38. TOPIC:(appraisal valence) AND TOPIC:( Extraversion)
39. TOPIC:(appraisal pleasantness) AND TOPIC:( Extraversion)
40. TOPIC:(appraisal goal) AND TOPIC:( Extraversion)
41. TOPIC:(appraisal aims) AND TOPIC:( Extraversion)
42. TOPIC:(appraisal implications) AND TOPIC:( Extraversion)
43. TOPIC:(appraisal motive) AND TOPIC:( Extraversion)
44. TOPIC:(appraisal agency)  AND TOPIC:( Extraversion)
45. TOPIC:(appraisal consequences)  AND TOPIC:( Extraversion)
46. TOPIC:(appraisal causality)  AND TOPIC:( Extraversion)
47. TOPIC:(appraisal outcome probability)  AND TOPIC:( Extraversion)
48. TOPIC:(appraisal goal conduciveness)  AND TOPIC:( Extraversion)
49. TOPIC:(appraisal urgency)  AND TOPIC:( Extraversion)
50. TOPIC:(appraisal expectations)  AND TOPIC:( Extraversion)
51. TOPIC: (appraisal coping potential) ANDTOPIC: (Extraversion)
52. TOPIC:(appraisal control) AND TOPIC:( Extraversion)
53. TOPIC:(appraisal power) AND TOPIC:( Extraversion)
54. TOPIC:(appraisal dominance) AND TOPIC:( Extraversion)
55. TOPIC:(appraisal adjustment) AND TOPIC:( Extraversion)
56. TOPIC:(appraisal normative significance) AND TOPIC:( Extraversion)
57. TOPIC:(appraisal internal standard compatibility) AND TOPIC:( Extraversion)
58. TOPIC:(appraisal external standard compatibility) AND TOPIC:( Extraversion)
59. TOPIC:(appraisal norms) AND TOPIC:( Extraversion)
60. TOPIC:(appraisal values) AND TOPIC:( Extraversion)
61. TOPIC:(appraisal moral values) AND TOPIC:( Extraversion)
62. TOPIC:(appraisal relevance) AND TOPIC:( Openness)
63. TOPIC:(appraisal novelty) AND TOPIC:( Openness)
64. TOPIC:(appraisal valence) AND TOPIC:(Openness)
65. TOPIC:(appraisal pleasantness) AND TOPIC:( Openness)
66. TOPIC:(appraisal goal) AND TOPIC:( Openness)
67. TOPIC:(appraisal aims) AND TOPIC:( Openness)
68. TOPIC:(appraisal implications) AND TOPIC:( Openness)
69. TOPIC:(appraisal motive) AND TOPIC:( Openness)
70. TOPIC:(appraisal agency)  AND TOPIC:( Openness)
71. TOPIC:(appraisal consequences)  AND TOPIC:( Openness)
72. TOPIC:(appraisal causality)  AND TOPIC:( Openness)
73. TOPIC:(appraisal outcome probability)  AND TOPIC:( Openness)
74. TOPIC:(appraisal goal conduciveness)  AND TOPIC:( Openness)
75. TOPIC:(appraisal urgency)  AND TOPIC:( Openness)
76. TOPIC:(appraisal expectations)  AND TOPIC:( Openness)
77. TOPIC: (appraisal coping potential) AND TOPIC: (Openness)
78. TOPIC:(appraisal control) AND TOPIC:( Openness)
79. TOPIC:(appraisal power) AND TOPIC (Openness)
80. TOPIC:(appraisal dominance) AND TOPIC:( Openness)
81. TOPIC:(appraisal adjustment) AND TOPIC: (Openness)
82. TOPIC:(appraisal normative significance) AND TOPIC:( Openness)
83. TOPIC:(appraisal internal standard compatibility) AND TOPIC:( Openness)
84. TOPIC:(appraisal external standard compatibility) AND TOPIC:( Openness)
85. TOPIC:(appraisal norms) AND TOPIC:( Openness)
86. TOPIC:(appraisal values) AND TOPIC:( Openness)
87. TOPIC:(appraisal moral values) AND TOPIC:( Openness)
88. TOPIC:(appraisal relevance) AND TOPIC:( Agreeableness)
89. TOPIC:(appraisal novelty) AND TOPIC:( Agreeableness)
90. TOPIC:(appraisal valence) AND TOPIC:( Agreeableness)
91. TOPIC:(appraisal pleasantness) AND TOPIC:( Agreeableness)
92. TOPIC:(appraisal goal) AND TOPIC:( Agreeableness)
93. TOPIC:(appraisal aims) AND TOPIC:( Agreeableness)
94. TOPIC:(appraisal implications) AND TOPIC:( Agreeableness)
95. TOPIC:(appraisal motive) AND TOPIC:( Agreeableness)
96. TOPIC:(appraisal agency)  AND TOPIC:( Agreeableness)
97. TOPIC:(appraisal consequences)  AND TOPIC:( Agreeableness)
98. TOPIC:(appraisal causality)  AND TOPIC:( Agreeableness)
99. TOPIC:(appraisal outcome probability)  AND TOPIC:( Agreeableness)
100. TOPIC:(appraisal goal conduciveness)  AND TOPIC:( Agreeableness)
101. TOPIC:(appraisal urgency)  AND TOPIC:( Agreeableness)
102. TOPIC:(appraisal expectations)  AND TOPIC:( Agreeableness)
103. TOPIC: (appraisal coping potential) ANDTOPIC: (Agreeableness)
104. TOPIC:(appraisal control) AND TOPIC:( Agreeableness)
105. TOPIC:(appraisal power) AND TOPIC:( Agreeableness)
106. TOPIC:(appraisal dominance) AND TOPIC:( Agreeableness)
107. TOPIC:(appraisal adjustment) AND TOPIC:( Agreeableness)
108. TOPIC:(appraisal normative significance) AND TOPIC:( Agreeableness)
109. TOPIC:(appraisal internal standard compatibility) AND TOPIC:( Agreeableness)
110. TOPIC:(appraisal external standard compatibility) AND TOPIC:( Agreeableness)
111. TOPIC:(appraisal norms) AND TOPIC:( Agreeableness)
112. TOPIC:(appraisal values) AND TOPIC:( Agreeableness)
113. TOPIC:(appraisal moral values) AND TOPIC:( Agreeableness)
114. TOPIC:(appraisal relevance) AND TOPIC:( Conscientiousness)
115. TOPIC:(appraisal novelty) AND TOPIC:( Conscientiousness)
116. TOPIC:(appraisal valence) AND TOPIC:( Conscientiousness)
117. TOPIC:(appraisal pleasantness) AND TOPIC:( Conscientiousness)
118. TOPIC:(appraisal goal) AND TOPIC:( Conscientiousness)
119. TOPIC:(appraisal aims) AND TOPIC:( Conscientiousness)
120. TOPIC:(appraisal implications) AND TOPIC:( Conscientiousness)
121. TOPIC:(appraisal motive) AND TOPIC:( Conscientiousness)
122. TOPIC:(appraisal agency)  AND TOPIC:( Conscientiousness)
123. TOPIC:(appraisal consequences)  AND TOPIC:( Conscientiousness)
124. TOPIC:(appraisal causality)  AND TOPIC:( Conscientiousness)
125. TOPIC:(appraisal outcome probability)  AND TOPIC:( Conscientiousness)
126. TOPIC:(appraisal goal conduciveness)  AND TOPIC:( Conscientiousness)
127. TOPIC:(appraisal urgency)  AND TOPIC:( Conscientiousness)
128. TOPIC:(appraisal expectations)  AND TOPIC:( Conscientiousness)
129. TOPIC: (appraisal coping potential) ANDTOPIC: (Conscientiousness)
130. TOPIC:(appraisal control) AND TOPIC:( Conscientiousness)
131. TOPIC:(appraisal power) AND TOPIC:( Conscientiousness)
132. TOPIC:(appraisal dominance) AND TOPIC:( Conscientiousness)
133. TOPIC:(appraisal adjustment) AND TOPIC:( Conscientiousness)
134. TOPIC:(appraisal normative significance) AND TOPIC:( Conscientiousness)
135. TOPIC:(appraisal internal standard compatibility) AND TOPIC:( Conscientiousness)
136. TOPIC:(appraisal external standard compatibility) AND TOPIC:( Conscientiousness)
137. TOPIC:(appraisal norms) AND TOPIC:( Conscientiousness)
138. TOPIC:(appraisal values) AND TOPIC:( Conscientiousness)
139. TOPIC:(appraisal moral values) AND TOPIC:( Conscientiousness)
